# Genomic and Transcriptome Analysis Reveals the Biosynthesis Network of Cordycepin in *Cordyceps militaris*

**DOI:** 10.3390/genes15050626

**Published:** 2024-05-15

**Authors:** Linshan Chai, Jianmei Li, Lingling Guo, Shuyu Zhang, Fei Chen, Wanqin Zhu, Yu Li

**Affiliations:** 1 Engineering Research Center of Chinese Ministry of Education for Edible and Medicinal Fungi, Jilin Agricultural University, Changchun 130118, China; lschai2019@163.com; 2 Liaoning Academy of Microbial Sciences, Chaoyang 122000, China; lijianmei5584@163.com (J.L.); lnwsw2013@163.com (L.G.); zsy6643@163.com (S.Z.); chenfei3033@vip.sina.com (F.C.); 18142101538@163.com (W.Z.)

**Keywords:** *Cordyceps militaris*, genome, population genome, transcriptome, cordycepin, metabolic pathway

## Abstract

*Cordycepin* is the primary active compound of *Cordyceps militaris*. However, the definitive genetic mechanism governing cordycepin synthesis in fruiting body growth and development remains elusive, necessitating further investigation. This study consists of 64 *C. militaris* strains collected from northeast China. The high-yielding cordycepin strain CMS19 was selected for the analysis of cordycepin production and the genetic basis of cordycepin anabolism. First, the whole-genome sequencing of CMS19 yielded a final size of 30.96 Mb with 8 contigs and 9781 protein-coding genes. The genome component revealed the presence of four additional secondary metabolite gene clusters compared with other published genomes, suggesting the potential for the production of new natural products. The analyses of evolutionary and genetic differentiation revealed a close relationship between *C. militaris* and *Beauveria bassiana*. The population of strains distributed in northeast China exhibited the significant genetic variation. Finally, functional genes associated with cordycepin synthesis were identified using a combination of genomic and transcriptomic analyses. A large number of functional genes associated with energy and purine metabolism were significantly enriched, facilitating the reconstruction of a hypothetical cordycepin metabolic pathway. Therefore, our speculation of the cordycepin metabolism pathway involved 24 genes initiating from the glycolysis and pentose phosphate pathways, progressing through purine metabolism, and culminating in the core region of cordycepin synthesis. These findings could offer fundamental support for scientific utilizations of *C. militaris* germplasm resources and standardized cultivation for cordycepin production.

## 1. Introduction

*Cordyceps militaris*, a model species of the genus *Cordyceps*, is widely distributed in terrestrial environments spanning cold and temperate climate zones, as well as tropical and subtropical regions, with the exception of Antarctica [1]. Due to its diverse array of biologically active compounds and unique pharmacological effects, it has been utilized for hundreds of years [2,3]. Designated as a new food resource by the Ministry of Health, *C. militaris* holds significant promise in healthcare applications. Since the 1860s, Basith Mumtaz [4] has successfully cultivated the fruiting bodies of *C. militaris* under artificial conditions. Over the subsequent decades, this cultivation method has advanced considerably, transitioning to large-scale factory production. However, market-available cultivated strains are currently characterized by limited genetic diversity, often resulting in similar products with different names. Moreover, the active ingredient content in most cultivated strains tends to be low, resulting in insufficient market competitiveness. Wild germplasm resources serve as invaluable repositories for breeding and offer abundant genetic diversity and potential materials for cultivating superior strains. However, due to the continuous deterioration of the ecological environment, the depletion of wild germplasm resources is accelerating, necessitating urgent reinforcement of efforts in the investigation, collection, research, and conservation of these invaluable resources.

Cordycepin, the primary active compound in *C. militaris*, has garnered significant research attention in recent years, particularly in the biochemical and molecular biology fields. For example, in terms of exogenous addition, the higher production of cordycepin in cultures utilizing xylose as the carbon source, along with significant enrichment in cordycepin synthesis according to transcriptome findings, has identified a 3′-AMP-related metabolic pathway for cordycepin synthesis [5]. Furthermore, amino acids significantly affect cordycepin synthesis. Supplementing substances, such as alanine, histidine, and rotenone, can activate biological pathways for both energy generation and amino acid conversion, thereby enhancing the production of cordycepin synthesis substrates [6]. Upon the completion of whole-genome sequencing of *C. militaris*, Xia et al. [7] discovered four genes (Cns1–Cns4) constituting the cordycepin biosynthesis gene cluster. These genes are physically connected and encode proteins with various conserved domains that facilitate cordycepin metabolism. Additionally, light is crucial for cordycepin synthesis [8]. Thannusak et al. [9] investigated the synthesis and metabolism pathways of cordycepin and carotenoids in response to light and observed the upregulation of the AMP pathway and 2-hydroxyglutaraldehyde-related genes under light conditions. The upregulation of genes related to the glycoses system metabolism was related to the biosynthesis of cordycepin and carotenoids, and appropriate light conditions promoted cordycepin synthesis. Nonetheless, despite numerous hypotheses regarding cordycepin biosynthesis, a comprehensive elucidation of these pathways remains elusive.

Hence, the objective of this study was to analyze the key genes involved in cordycepin anabolism and their metabolic pathways and to achieve scientific and efficient cordycepin production. In this study, genomic and comparative analyses were conducted on the wild-type strain CMS19, which exhibited promising traits obtained through early-stage collection, cultivation, and domestication. This study aimed to (1) obtain technical parameters for cordycepin production by investigating its growth patterns; (2) elucidate the genome composition and structure of CMS19 at the genomic level; (3) explore the genetic diversity, structure, and differentiation in the northeastern population through the population genome analysis, identifying functional genes related to cordycepin synthesis; and (4) analyze the genetic basis of cordycepin synthesis metabolism during fruiting body growth through transcriptome sequencing. The results provide fundamental data supporting cordycepin anabolism and lay the foundation for further endeavors in breeding new varieties, commercial cultivation, and acquisition of high-yielding cordycepin strains through metabolic regulation techniques.

## 2. Materials and Methods

### 2.1. Strains and Cultivation

A total of 64 strains, comprising 48 wild strains and 16 cultivated strains were used in this study (Table S1), which was collected from northeast China, and maintained at Liaoning Microbial Strain Maintenance Center. Cultivation evaluation was conducted on 64 strains, and the excellent strain CMS19 was subjected to monitoring of changes in cordycepin at different growth and development stages. The process of cultivation used the following methods [10]: First, strains were cultured at 20 °C on potato dextrose agar. Then, mycelium was inoculated into liquid culture medium (10 g potato flour, 20 g dextrose, 5 g peptone, 0.2 g KH_2_PO_4_, 0.1 g MgSO_4_ in 1 L water) to prepare liquid spawn. A total of 30 g wheat with 45 mL of prepared liquid medium were added to each box, which was then sealed with plastic and sterilized in autoclave for 20 min at 121 °C. The sterilized substrates were inoculated with 5 mL liquid spawn in each bottle. They were cultured at 20 °C in darkness until the substrates were covered by white mycelia, these bottles were then transferred to a culture room at 22 °C under scattered sunlight of 500 lx for 14 h and darkness for 10 h with humidity of 60–80% to produce fruiting bodies.

### 2.2. Determination of Cordycepin

Cordycepin and adenosine were determined by high-performance liquid chromatography (HPLC, Waters 2695, Waters Corp., Milford, MA, USA) in which the mobile phase was acetonitrile/ultrapure water (5:95, *v*/*v*). Elution was performed at a flow rate of 1.0 mL/min on Agilent Luna 5μ C18(2) 100 A (250 mm × 4.6 mm) at 35 °C, using a UV detector (Waters 2498, MA, USA) with a wavelength of 260 nm. Empower ^TM^ 3 software (v1.66.43) was used to calculate the content of cordycepin and adenosine.

### 2.3. Genomic DNA Extraction and Purification

CMS19 is a wild strain characterized by high resistance, yield, and cordycepin content, which was collected from northeast China. Mononuclear CMS19 strain was obtained via protoplast isolation as in a previous study [11], the obtained monokaryou was used for de novo genome sequencing. A total of 64 strains was used in the whole-genome resequencing (Table S1). Mycelia were cultured at 25 °C on PDA medium for 15 d, the mycelial was harvested, then frozen in liquid nitrogen to aid in grinding the sample. The genomic DNA was extracted using the NuClean Plant Genomic DNA Kit (CWBIO, Beijing, China) according to the instructions, and its integrity, purity, and concentration were assessed using 0.8% agarose gel electrophoresis, Nanodrop 2000, and Qubit, respectively.

### 2.4. De Novo Genome Sequencing, Assembly, and Annotation

The genome of CMS19 strain was sequenced using the PacBio R II long-read sequencing and Illumina HiSeq PE150 platform with the 20 kb and 350 bp insert size, respectively [12]. De novo assembly was conducted using SMRT Link V5.0.1 software and SOAP de novo v2.04 software (parameters: - d 1, - m 3, - r, - u, - f, - k 55) [13] with PacBio long reads. Then, two rounds of polariting with Illumina short reads were optimized and filled using krskgfv1.2 and gapclose v1.12 software [14]. The integrity of final assembly was assessed by core eukaryotic gene mapping approach (CEGMA) and benchmarking universal single-copy ortholog (BUSCO) [15]. The genome of CMS19 was uploaded to the figshare database (https://figshare.com/articles/dataset/_i_Cordyceps_militaris_i_CMS19/25592604 (accessed on 20 April 2024)).

To systematically annotate genes in the assembled genome, we integrated gene model predictions from de novo and homolog methods. Augustus (v3.4.0) [16] were used for de novo gene predictions. For homolog gene-based prediction, tBLASTn and Gene wise were used to conduct ab initio prediction with *Fusarium graminearum* as reference species [17]. Subsequently, the predicted genes were conducted using blastp (BKAST 2.2.28+) [18] with databases such as Non-Redundant Protein Sequence Database (Nr), Nucleotide Sequence Database (Nt), Gene Ontology (GO), Clusters of Orthologous Groups for eukaryotic complete genomes (KOG), Kyoto Encyclopedia of Genes and Genomes (KEGG), InterPro, and SwissProt to obtain functional descriptions of genes. The integrated results were used for subsequent analyses. Repetitive sequence was predicted by RepeatMasker (v4.3.7) [19]. The tRNA and rRNA were predicted by tRNAscan-SE (v1.3.1) [20] and Barrnap (v0.4.2) software [21]. Non-coding RNAs, such as small nuclear RNA (snRNA) and microRNA (miRNA), were annotated using the Rfam [22]. GO [23] annotation was used by hmmer2go (v0.17.9). KEGG [24] annotation was used by the blast algorithm (blast x/blastp 2.11.0, e: le-5) to compare all genes with the KEGG gene database, and analyze the biological pathways that genes utilized. The gene clusters associated with secondary metabolic biosynthesis of CMS19 was predicted using antiSMASH database as default parameter settings (https://fungismash.secondarymetabolites.org (accessed on 12 April 2024)); then, the CM01 and ATCC34164 of C. milittaris genome from NCBI database were used to compare predictions of secondary metabolism of these three strains.

### 2.5. Whole-Genome Resequencing and Data Analysis

All 64 strains were used in the whole-genome resequencing. DNA libraries (350 bp) for Illumina sequencing by HiSeq xTen platform were constructed for each accession according to the manufacturer’s specifications (Majorbio technologies, Beijing, China). Raw reads were filtered based on the following criteria [25,26]: pair-end reads with >10% ‘N’ bases; reads on which more than 50% of the bases have a quality score less than 20 (Phred-like score) were used to obtain clean reads. All clean reads for each accession were mapped to reference genome using the MEM algorithm of Burrows–Wheeler Aligner (bwa-mem2 v2.2) [27]. SNPs and indels within 64 accessions were called using the HaplotypeCaller module in GATK (v3.8) [28]. PCA used the smart pca program in the Eigensoft software (v5.0.1) [29]. Phylogenetic analysis used SNPhylo [30] to extract SNPs from homologous regions.

### 2.6. Orthology Comparisons and Population Phylogenetic Analysis

The evolution and genetic differentiation of *C. militaris’* genome evolution was analyzed alongside four genomes available from NCBI, including *B. bassiana*, *F. graminearum*, *Metarhizium anisopliae*, and *Ophiocordyceps sinensis,* and *Neurospora crassa* was used as the outgroup. Subsequently, a phylogenetic tree was constructed using maximum likelihood (ML tree) with 100 bootstraps by MEGA software (v10.1.3) [31]. Population genetic structure and phylogenetic analysis of 64 strains used faststructure software [32] and MEGA software (v10.1.3) (bootstraps 200) [S31], respectively.

### 2.7. Linkage Disequilibrium Analysis and Selection Elimination Analysis

Two populations consisting of high (group1 > 3.00 mg/g) and low (group2 < 3.00 mg/g) cordycepin strains (Table S1) were designated, with r2 employed as a metric for the decay of linkage disequilibrium (LD). The r2 value, reflecting the degree of statistical and genetic correlation between loci (0 < r2 < 1), was determined between SNPs using [33]. The Fst value between the two populations was computed using PoPoolation2 software (v4.2) [34], while the vcftools software calculated the Pi values of the populations. Subsequently, the selected regions were determined based on a combination of the *Fst* (size 20000, step 2000) and *Pi* (20000, step 2000). Finally, Blast2GO software (v2.5) [35] was used to perform GO enrichment analysis of the candidate genes.

### 2.8. Transcriptome Sequencing and Differential Expression Analysis

According to the developmental characteristics and Cordycepin content of CMS19 strain; therefore, T30d was characterized as low cordycepin level group, and T50d and T70d as high cordycepin level groups. The transcriptome sequencing was performed at these three stages. The total RNA was extracted through applying the TRIzol reagent. Subsequently, an Agilent Technologies 2100 Bioanalyzer was evaluate the integrity and quality. Three tubes of the total RNA were extracted as biological replicates for each stage. Following the manufacturer’s standard protocols, a total of nine libraries were established. The paired-end sequencing was then conducted on all these libraries using the Illumina HiSeq 2500 platform (Majorbio Technologies).

Clean data were obtained by processing the raw machine data using fastp filtering software, which removed adaptors, reads with an unknown base content of >10%, and low-quality reads. The clean reads were aligned to the reference genome sequence using Bowtie2 software (v2.5.2) [36]. The “cor” function of R software (v4.1.1) was used to calculate the Pearson correlation coefficients among samples and the gene expression levels for each sample were determined using RSEM. The DEGseq software (v4.1.1) [37] was used to identify differentially expressed genes.

Differentially expression genes (DEGs) were functionally annotated for GO [23] and KEGG [24], and enrichment analysis was conducted using the phyper function in R software based on the annotation results. The *p*-values (*p* < 0.05) were then subjected to FDR correction, and those with *Q*-values < 0.05 were considered significantly enriched. The expression data for these samples were deposited in the Figshare database (https://figshare.com/articles/dataset/_i_Cordyceps_militaris_i_CMS19_Transcriptome_data/25594245 (accessed on 12 April 2024)).

### 2.9. qRT-PCR Validation

The qRT-PCR used the same RNA samples with the transcriptome sequencing. Three biological replicates were used for these treatments and were subsequently reverse-transcribed into cDNA. Following the protocol outlined in the Transgene biotech fluorescence quantification kit, thirteen selected differential gene data associated with cordycepin production were identified using the ABI SteponePlus instrument. The reference gene was β-actin (β-actin F: GGAGAAGATTGGCATCACACA and β-actin R: GAAGAGCGAAACCCTCGTAGA). After completion of the experiment, gene expression analysis was conducted using the 2^−ΔΔCt^ method [38].

## 3. Results

### 3.1. Analysis of Cordycepin Production Regularity

**Figure 1:**
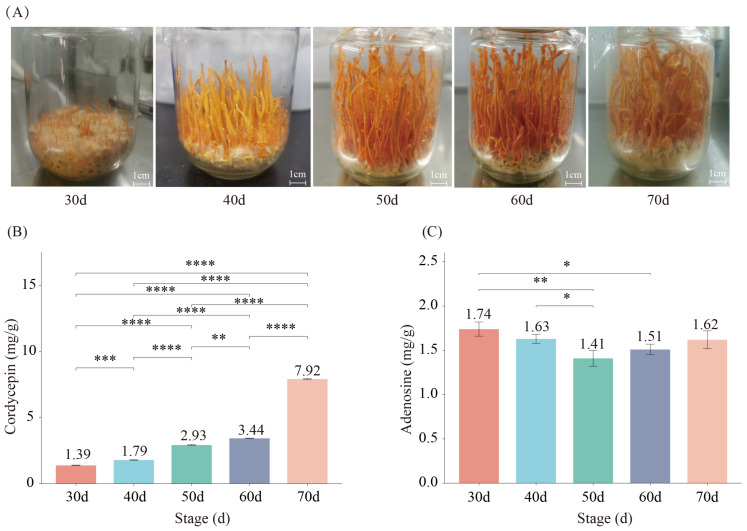
Content of cordycepin and adenosine at different growth stages in *C. militaris*. (**A**) Development of fruiting bodies at different stages; (**B**) content of cordycepin at different stage; (**C**) content of adenosine at different stage. Note: * represent *p* value < 0.05; ** represent *p* value < 0.01; *** represent *p* value < 0.001; **** represent *p* value < 0.0001.

To investigate the changes in cordycepin content throughout the various stages of fruiting body growth and to analyze its genetic mechanisms, a quantitative analysis of cordycepin content within the fruiting bodies of the wild strain CMS19 was conducted during five distinct periods of artificial cultivation: 30 d (T30d), 40 d (T40d), 50 d (T50d), 60 d (T60d), and 70 d (T70d) (Figure 1A). The results indicated the following cordycepin content for each period were significantly different (*p* < 0.01): T30d (1.39 mg/g), T40d (1.79 mg/g), T50d (2.93 mg/g), T60d (3.44 mg/g), and T70d (7.92 mg/g) (Figure 1B). Adenosine content significantly decreases from 30 d to 50 d (*p* < 0.05), following a slight increase. During the development of fruiting bodies, cordycepin content exhibited considerable variation, increasing by 5.96 times from 30 d to 70 d. Conversely, adenosine content showed a trend of first decreasing and then increasing; it was highest at 30 d, then lowest at 50 d, gradually increasing afterwards. This indicated that adenosine as a substrate for cordycepin synthesis was undergoing dynamic changes and could be the most active in cordycepin synthesis at 50 d (Figure 1C).

### 3.2. Genome Component of C. militaris

To investigate the genetic mechanisms underlying the cordycepin synthesis across different time periods, whole-genome sequencing on CMS19 obtained 26.1 Gb data, with 3,775,077 read counts. The final assembled genome size was 30.96 Mb, comprising 8 contigs, with the longest contig measuring 7,648,282 bp, N50 value of 4,735,179 bp, and GC content of 52.69%. Augustus software was used for ab initio prediction, contributing to the prediction of 9781 protein-coding genes, which accounted for 48.23% of the genome size. The average length of the predicted protein-coding genes was 1527 bp, with an average of 2.84 exons per gene and an average exon length of 537 bp (Table 1). CEGMA and BUSCO tests yielded scores of 96.37% and 95.8%, respectively. These results indicated the high integrity of the CMS19 genome assembly, providing a robust foundation for further investigation.

### 3.3. Genome Annotation

These genes were further annotated in the NR, KEGG, KOG, GO, and Swiss-Prot databases, with annotations of 9650 (98.66%), 6510 (66.56%), 5120 (52.35%), 6034 (61.69%), and 6412 (65.56%) genes, respectively (Table S2). The total repeat content was 1,317,103 bp, representing 4.25% of the genome size. Retroelements occupied 545,114 bp and accounted for 1.76% of the genome size. Among the various transposable element species, the long terminal repeats (LTRs) comprised the largest portion, spanning a length of 524,914 bp, accounting for 1.70% of the genome size. Both the GYPSY/DIRS and Ty1/Copia transposons totaled 227,612 bp, accounting for 0.92% of the genome. Long interspersed nuclear elements (LINEs) encompassed 20,139 bp, constituting 0.07%. Additionally, the microsatellites and simple repeat sequences were predicted to have lengths of 2478 bp and 16,708 bp, respectively, accounting for 0.01% and 0.05% of the genome, respectively (Figure S1).

### 3.4. Prediction of the Secondary Metabolite Synthesis Genes

We annotated 32 secondary metabolite gene clusters in the *C. militaris* genome across 6 longer contigs. The numbers of secondary metabolite synthesis gene clusters on each chromosome were six, nine, four, three, five, and five. The types of secondary metabolite synthesis gene clusters varied among individuals. These included eight T1PKS-type gene clusters, seven NRPS-type gene clusters, five NRPS-like gene clusters, four terpene-type gene clusters, two TIPKS/NRPS-type gene clusters, four NRPS/T1PKS-type gene clusters, one indole-type gene cluster, and one fungal RIPP-type gene cluster, which were categorized based on the enzymes encoding secondary metabolite synthesis genes (Table S3). Moreover, database annotations indicated high homology between the synthetic gene clusters of substances, such as squalestatin S1, dimethylcoprogen, phomasetinp, homasetin, ustiloxin, neosartorin, clavariant acid, emericellamide A/emericellamide B, and viridoxin, and those of known species (Table S4).

We predicted the secondary metabolites of CMS19 compared with the other two *C. militaris* genomes (ATCC34164 and CM01) that were obtained from NCBI. The analysis revealed 28 secondary metabolites predicted by both CM01 and ATCC34164 genomes, which was four fewer than the predictions derived from the CMS19 genome. Subsequently, gene cluster predictions from each genome were screened to identify significant differences in the number of predicted gene clusters among the three genomes (Table 2). Notably, three substance synthesis gene clusters, ustiloxin B, fumosorinone, and emericellamide A/emericellamide B, were exclusively predicted in the CMS19 genome, with matching rates of 31%, 83%, and 60%, respectively. The prediction of secondary metabolite gene clusters in *C. militaris* demonstrated its potential for producing a variety of new natural products.

### 3.5. Genome Mutation

To investigate the genetic diversity and population structure of *C. militaris* populations in northeast China, we conducted whole-genome resequencing of 64 samples comprising 48 wild and 16 cultivated strains. In total, 630 million high-quality clean reads were obtained (Table S5). Each strain had an average sequence coverage over 95.1% and a Q30 value exceeding 93.20%. In total, 681,562 high-quality SNPs and 229,221 indels were detected. Among them, synonymous mutations located in the gene-coding region (CDS region) of the SNP comprised the largest proportion of the overall strain variation, accounting for approximately 40.16%, and non-synonymous mutations in the CDS region accounted for 13.81%. In addition, an average of approximately 22.81% of upstream SNPs, 15.35% of downstream SNPs, 1.07% of intergenic SNPs, and 4.52% of intronic SNPs were identified (Table S6). In the context of indels, all the strains exhibited homozygous mutations without heterozygous mutations; most indels were observed in the upstream and downstream regions of the genes. Specifically, approximately 44.75% were identified in the upstream region, whereas approximately 28.98% were located in the downstream region of the gene. This information provides a novel genetic resource for the biology and breeding of *C. militaris*.

### 3.6. Analysis of Phylogenetic Relationships and Population Structure

**Figure 2:**
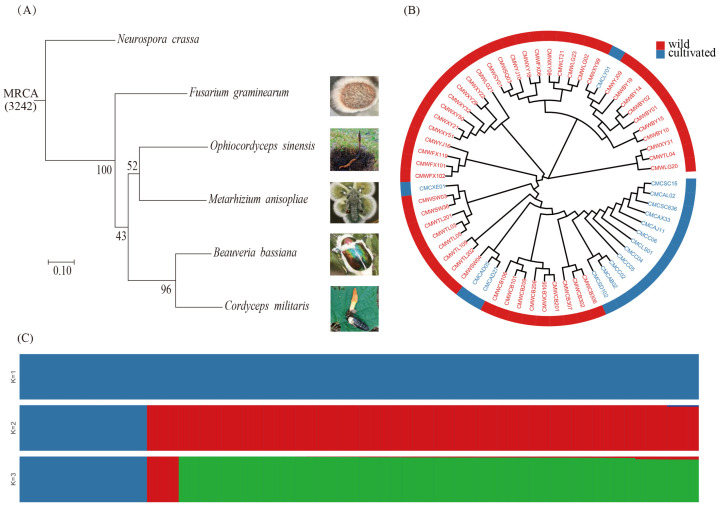
Phylogenetic relationships and population structure of *C. militaris*. (**A**) Phylogenetic relationships based on single-copy genes; (**B**) phylogenetic relationships based on SNPs; (**C**) population structure.

A total of 3242 single-copy orthologs were identified using the gene family analysis. Subsequently, a phylogenetic tree was constructed using maximum likelihood with N. crassa as the outgroup (Figure 2A). These results indicated a close relationship between *C. militaris* and *B. bassiana*. Among the selected species, *C. militaris* exhibited the most recent differentiation, and *F. graminaearum* exhibited the earliest differentiation. The result was consistent with the Sung report [39], which used 162 taxa to analysis the phylogenetic relationships of cordyceps based on five to seven loci. Results support the existence of three clavicipiaceous clades and show *Fusarium* as a basal clade in Hypocreales, and Cordyceps close relationship with *Bassiana* within the family Cordycipitaceae.

To investigate the genetic diversity and population structure of *C. militaris* populations in northeast China, we conducted a population genetic analysis of 48 wild (red) and 16 cultivated (blue) strains base on resequencing databases. The phylogenetic results revealed most of the wild isolates group together, and most of the cultivated strains group together, while a few cultivated strains mixed with the wild strains group (Figure 2B). Notably, the four cultivated strains were grouped into various subgroups in the wild region. Specifically, CMCLY01, collected in Liaoyang, clustered with strains from the Xiuyan area of Anshan, whereas the remaining three cultivated strains clustered alongside wild strains from Shenyang and Tieling.

Population structure analysis yielded results that were consistent with the phylogenetic findings (Figure 2C). When the ancestor was set to *K* = 2, the wild strains (red) and 12 cultivated strains (blue) initially diverged, forming two groups. At *K* = 3, 3 groups of strains emerged: 12 cultivated strains (blue), 3 wild strains, CMWCB302, CMWCB306, and CMWCB307 (red), and other strains (green). Notably, the red group of three wild strains were collected far from the other wild strains and exhibited distinct geographical regional differences. The results demonstrated that throughout the phylogenetic evolutionary process, differentiation between the wild and cultivated strains was evident.

### 3.7. Analysis of the Selective Elimination of Functional Genes Related to Cordycepin Synthesis

**Figure 3:**
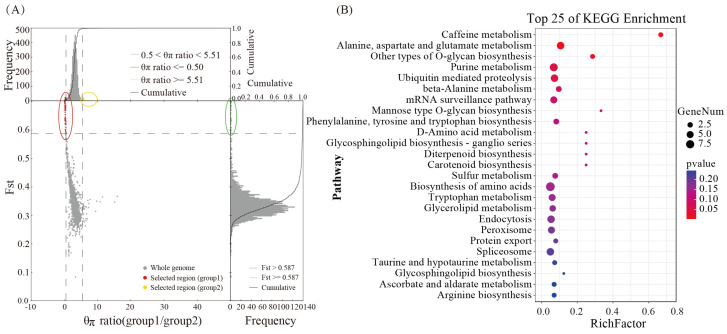
Selecting elimination analysis to explore functional genes related to cordycepin metabolism. (**A**) Analysis of selection and elimination of high- and low-cordycepin groups. (**B**) KEGG enrichment of selected genes.

To investigate individual differences in cordycepin synthesis among various strains and to identify the functional genes associated with cordycepin synthesis, strains with varying cordycepin contents were categorized based on previous research (Table S1). Subsequently, the functional genes linked to cordycepin synthesis were identified through group selection and elimination analysis, resulting in the identification of 331 significantly correlated candidate genes (Figure 3A). The KEGG enrichment analysis indicated that these functional genes were predominantly related to caffeine, alanine, aspartate, and glutamate metabolism, O-glycan biosynthesis and purine metabolism (Figure 3B). In addition, a substantial number of protein-coding genes related to amino acid metabolism, RNA transport, and carotenoid metabolism were identified, potentially influencing raw material supply, fruiting body color, and cordycepin production.

### 3.8. Transcriptome Studies of Cordycepin Anabolism

**Figure 4:**
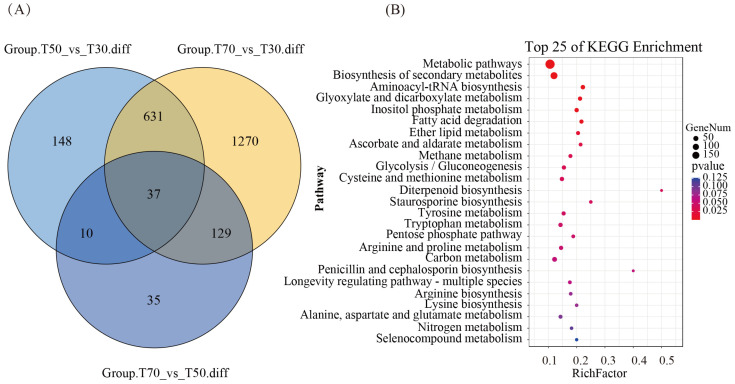
Transcriptome studies of cordycepin anabolism. (**A**) Comparison of differentially expressed genes; (**B**) KEGG enrichment of upregulated genes.

Differential expression analysis of transcriptome revealed that these were 826 (up:408, down:418) and 2067 (up:863, down:1204) DEGs at T30d compared to T50d and T70d, respectively, and 211 (up:57, down:154) DEGs between T50d and T70d (Figure 4A). Notably, compared with T30d, there were 320 genes that were consistently upregulated, whereas 346 were downregulated across both T50d and T70d stages. Only 37 genes were differentially expressed in each comparison group. Then, we compared 331 genes from selective elimination analysis with their expression levels in the transcriptome, and identified 331 genes with varying degrees of differential expression, including 156 upregulated and 175 downregulated genes. The enrichment pattern of upregulated genes was consistent with a selective elimination trend; these genes would provide assistance for the study of the cordycepin synthesis pathway.

The transcriptome functional enrichment analysis showed that metabolic pathways, biosynthesis of secondary metabolites, aminoacyl tRNA biosynthesis, glyoxylate, and dicarboxylate metabolism were enriched, and various amino acid synthesis and metabolism were all enriched. Regarding selecting upregulated genes of selective elimination, these differentially expressed genes presented the significant enrichment of glycolysis, the pentose phosphate pathway, and purine metabolism among stages with low- and high-cordycepin content (Figure 4B). Additionally, the substantial enrichment of amino acid metabolism indicated a positive impact on the cordycepin synthesis [6]. Furthermore, focusing on genes of the adenosine synthesis pathway, expressions of adenylate kinase (g5449), adenosine kinase (g3376), adenylosuccinate synthetase (g1229), and adenosine deaminase (g2847, g3195) were observed in the high-cordycepin stage. In particular, the high expression of adenylosuccinate synthetase may facilitate the provision of adenosine and other precursor substances for the cordycepin synthesis [6,7].

### 3.9. Prediction of Hypothetical Metabolic Pathways of Cordycepin

**Figure 5:**
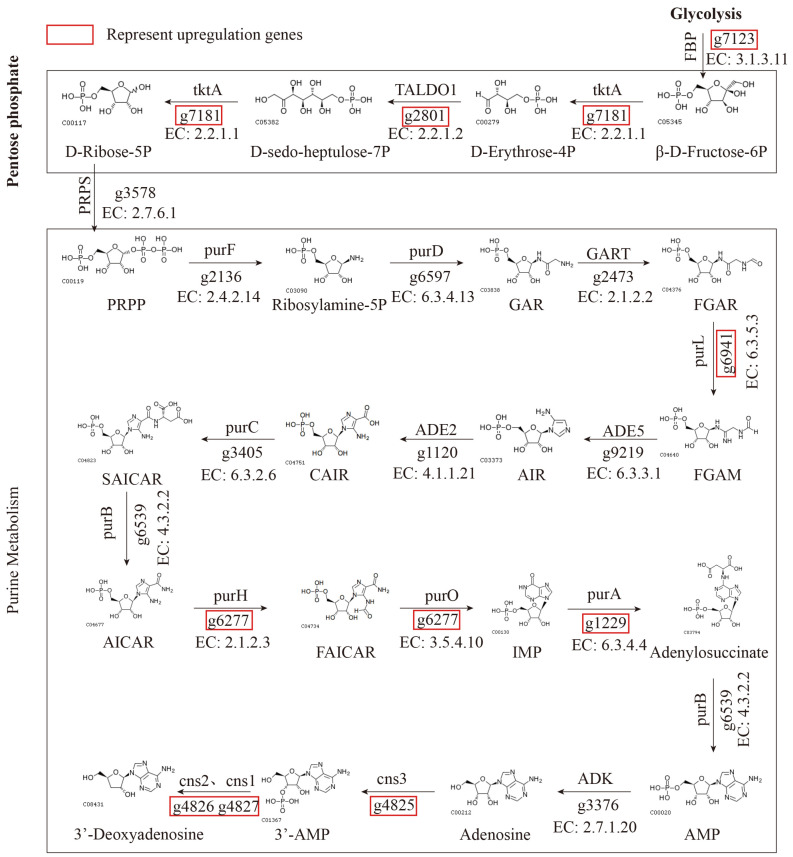
Hypothetical metabolic pathways of cordycepin in *C. militaris*.

Our study identified the metabolic pathway involving adenosine as purine metabolism. Hence, we proposed purine metabolism as the endpoint of cordycepin synthesis, predicting the metabolic pathway and associated functional genes; therefore, we speculated that the metabolic pathway comprising 21 genes could represent the cordycepin metabolism pathway (Figure 5, Table S7). Integrating the above genomic and transcriptome enrichment analyses, we suggested that the cordycepin synthesis could begin with Glycolysis, yielding D-Ribose-5P for the pentose phosphate pathway. In the pentose phosphate pathway, we annotated a pathway through which five enzyme genes catalyzed the generation of the important starting substance PRPP, required for purine metabolism. All five genes were upregulated and four were significantly upregulated. After the involvement of PRPP in the purine metabolism, a pathway catalyzed by 15 prominently upregulated enzyme genes was annotated, to the formation of adenosine. Compared to 30 d, the genes significantly upregulated at 50 and 70 d were g7123 (620.0, 1465.7, 1783.0), g7181 (2053.3, 4992.0, 7730.7), g2801 (1732.3, 5624.4, 7878.0), g6941 (1304.7, 2753.0, 3463.3), g6277 (1840.0, 4921.7, 7097.3), and g1229 (514.3, 1212.0, 2100.3) in these cordycepin synthesis pathways. In addition, g6597, g6539, and g3376 all showed high levels of upregulation. Adenosine, commonly recognized as a precursor for cordycepin synthesis, can generate cordycepin through the action of Cns1–4 [7]. Our study further examined the expression levels of these four following genes: Cns 1 (g4827), Cns 2 (g4826), Cns 3 (g4825) and Cns 4 (g4824), across different stages of the fruiting body development. The transcriptional levels of Cns 1 (T30d: 2170.7, T50d: 7232.0, T70d: 7073.3) and Cns 2 (T30d: 4154.0, T50d: 9606.0, T70d: 8580.0) were significantly upregulated, and Cns 3 (T30d: 1724.0, T50d: 3838.0, T70d: 3431.0) was slightly increased; however, in Cns 4 (T30d: 1749.3, T50d: 1655.0, T70d: 1838.7) there was no difference. Among the three stages, the expression levels of all Cns1, Cns2, and Cns3 were the highest at 50 d, and slightly decreased at 70 d compared to 50 d. This revealed that these genes related to cordycepin synthesis were continuously expressed at high levels from 50 d to 70 d.

### 3.10. Differential Gene Expression Measured Based on qRT-PCR

Some studies have shown that the Cns1~Cns4 genes were key genes in the synthesis of cordycepin and some genes related to adenosine metabolism pathways were also involved in cordycepin biosynthesis [6,7,11]. Therefore, in the qRT-PCR project, we referred to above research to screen 13 genes that were believed to be involved in cordyceps synthesis (Table 3), to verify the accuracy of transcriptome sequencing and further detect the expression patterns of these genes. Overall, most of the genes showed a similar result to the RNA-Seq with minor differences, but some showed discrepancies. This indicates that RNA-seq data can be applied to transcriptome analysis.

## 4. Discussion

*C. militaris*, a model species within the cordyceps genus, has emerged as a substantial industry in China, offering significant economic and health benefits as a substitute for *O. sinensis* [40]. Cordycepin, a principal component of its efficacy [41] has been the subject of considerable research on culture condition optimization, active ingredient analysis, and pharmacological effects, providing valuable insights for this study [42,43]. However, the existing germplasm resources have failed to meet production demands, demonstrating the importance of exploring wild resources to acquire desirable characteristics. Based on previous germplasm collection and evaluation efforts, a wild *C. militaris* strain, CMS19, exhibiting high yield and cordycepin content, has been identified as an excellent resource for commercial *C. militaris* enhancement. Furthermore, this study determined the optimal culture time for this strain to be 70 d, based on production requirements, providing crucial data for subsequent production applications.

Research on cordycepin anabolism in *C. militaris* is a prominent focus. Recent studies by experts and scholars have extensively explored the optimization of culture conditions, exogenous additives, and the use of omics tools. While NCBI has published genomic data for three *C. militaris* strains [44,45], only one strain, originating from Canada, assembled to the chromosome level. The CMS19 strains used in this study were sourced from Liaoning province, China, and exhibited a slightly higher cordycepin content than the current main varieties, suggesting adaptive evolution in long-term field environments. This study sequenced the whole genome, contributing to the assembly of 8 contigs, which contain 9781 genes. This genome was aligned more closely with the genetic characteristics of *C. militaris* in China and serves as a valuable reference for future studies. A total of 5 genomes from NCBI were used for orthology comparisons and phylogenomic placement, a total of 3242 single-copy orthologs were identified using the gene family analysis, and *C. militaris* has a closer genetic relationship with *B. bassiana*. *F. graminearum* exhibited the earliest differentiation and *C. militaris* exhibited the most recent differentiation in biological evolution, this is consistent with previous research [46]. Previously sequenced genes from *Cordyceps*, *Ophiocordyceps* and related genera were predicted to have over 30 secondary metabolites [47,48,49]. Secondary metabolites in CMS19 were predicted, revealing the presence of 32 secondary metabolite gene clusters within the genome. Concurrently, both the published genomes CM01 and ATCC34164 predicted 28 secondary metabolites [45], highlighting 4 additional potential secondary metabolites in CMS19, including ustiloxin B, fumosorinone, and emericellamide A/B. This prediction of secondary metabolite gene clusters demonstrated the capacity of CMS19 to produce a range of novel products, offering a bioinformatics foundation for discovering and exploiting valuable new metabolites in *C. militaris*. Moreover, this suggests the possibility of uncovering valuable metabolites in the future.

The SNP variant data derived from population genomics analysis play a crucial role in elucidating genetic differentiation and key functional genes among populations of edible and medicinal mushrooms that can be common across various species such as *Lentinula edodes* and *Ganoderma lingzhi* [50,51]. In this study, we performed population genomic analysis of 64 wild and cultivated *C. militaris* strains from northeast China. Our findings demonstrated substantial genetic diversity within the strain population. Notably, significant genetic differentiation was observed between the wild and cultivated populations; most cultivated strains (12 out of 16) could be distinguished from the population. The remaining four cultivated strains exhibited close genetic proximity to the wild strains. The results suggest either that cultivated strains have been domesticated multiple times, potentially from different wild populations and/or that human cultivation could have allowed infiltration of germplasms into natural populations. Furthermore, there was geographical genetic diversity within the wild populations, with the strains from Jilin and Liaoning showing early divergence. This abundant genetic diversity serves as a foundational resource for the utilization of *C. militaris* germplasm.

In recent years, research on cordycepin synthesis and metabolism has gained considerable attention [52,53]. The gene expression profiles of *C. militaris* at different growth and development stages show rich differences, there are also significant differences in secondary metabolites such as cordycepin [54,55], and cordycepin is closely associated with glucose accumulation [56]. In this study, we found that there were significant differences in cordycepin content in the fruiting bodies at different developmental stages, and the trend gradually increased. Studying the changes in gene expression patterns at different stages and exploring genes related to cordycepin synthesis could deepen the understanding of the synthesis pathway of cordycepin. Through the population selection elimination analysis and expression level analysis of high- and low-cordycepin content groups, we identified significantly enriched metabolic processes including caffeine metabolism, glycolysis, purine metabolism, and the pentose phosphate pathway. Given the importance of adenosine, particularly deoxyadenosine, as a precursor for the cordycepin synthesis [57,58], we suggested that the cordycepin synthesis could begin with the Glycolysis and that the purine metabolism were the primary process for cordycepin production. Furthermore, we suggested the involvement of the pentose phosphate pathway in generating the PRPP necessary for purine metabolism. Therefore, we hypothesized that the pentose phosphate pathway may play a role in cordycepin synthesis. Moreover, we suggested that the cordycepin synthesis should initiate the glycolysis and the pentose phosphate pathway, supplying essential precursors for cordycepin production, consistent with prior research [59]. Adenylosuccinate synthetase (ADL) and 5′-nucleotidase (NE5T) pay important roles in this metabolism. After the PRPP pathway, inosine monophosphate (IMP) and L-Asp are converted to N6-(1,2-dicaboxyethyl)-AMP under the catalysis of adenylosuccinate synthase (ADL). Then, AMP and adenosine are formed under the catalysis of adenylosuccinate synthase (ADL) and 5′-nucleotidase, respectively [7,54]. In the comparison of differentially expressed genes in the transcriptome, the adenylosuccinate synthetase gene (g1229) was upregulated by 2.35 times at 50 d and 3.30 times at 70 d, respectively. Additionally, the expression level of the 5′-nucleotidase gene (g1923) increased by 2.22 times and 5.38 times (T30d 475.0, T50d 1054.7, T70d 2557.7). There are four widely recognized genes related to cordycepin synthesis, Cns1-Cns4, which encode oxidoreductase, metal ion-dependent phosphohydrolase, ATP-dependent phosphotransferase, and ABC-type transporters [7]. Our study conducted expression level analysis of these four genes and observed the upregulation of the Cns1–3 genes, with Cns1 and Cns2 significantly upregulated, Cns3 slightly upregulated in both 50 d and 70 d, and the Cns4 gene remaining unchanged. Compared to 30 d, the expression levels of Cns1, Cns2, and Cns3 were the highest at 50 d (T50 vs. T30 Cns1: fc 3.33, Cns2: fc 2.31 and Cns3: fc 2.23) and slightly decreased at 70 d (T70 vs. T30 Cns1: fc 3.26, Cns2: fc 2.07 and Cns3: fc 1.99). This revealed that these genes related to cordycepin synthesis had continuous high-level expression from 50 d to 70 d; therefore, the accumulation of cordycepin lagged behind gene expression, when the cordycepin content was higher at 70 d. These results validate our hypothesis about the cordycepin synthesis pathway.

In addition, we identified the gene-encoding proteins related to oxidation–reduction, glutamate and glutamine metabolism, alanine metabolism, carotenoid metabolism, and RNA transport. These genes play direct or indirect roles in cordycepin synthesis by facilitating energy delivery and interconversion-related precursors or substrates within the cordycepin anabolic pathways. Xia et al. [7]. reported that pentostatin, encoded by Cns3, regulates cordycepin synthesis by inhibiting ADA activity and serves as a key factor in cordycepin accumulation. Transporters play crucial roles in pentostatin transport. Therefore, the significant selection pressure observed for transport-related proteins in this study was closely linked to the strains exhibiting high-cordycepin content, which was consistent with previous findings. Moreover, amino acids significantly affect cordycepin content [60,61]. Oh [56] conducted a GC-MS analysis of *C. militaris* fruiting bodies, revealing a correlation between cordycepin biosynthesis and glutamine and glutamate metabolism. This study also identified a significant selection of genes associated with glutamate and glutamine metabolism along with functional genes related to alanine metabolism, further suggesting their correlation with cordycepin content. In addition, this study enriched the carotenoid-related genes [62]. Studies have indicated a close association between carotenoid, color, and cordycepin production in *C. militaris* [63]. Moreover, the relevant literature suggests that during cordycepin fermentation, vitamin content can significantly enhance the cordycepin yields [64]. Hence, the carotenoid-related functional genes identified in this study may further affect cordycepin accumulation by medulating vitamins. However, the anabolic process of cordycepin can be a complex metabolic network, in which the activation of one metabolism may influence others. A deep understanding of these metabolic changes may contribute to a comprehensive understanding of the regulatory mechanisms underlying cordycepin synthesis. Therefore, it is necessary to include metabolomics in future research.

## 5. Conclusions

In this study, high-quality whole-genome sequences of high-yielding cordycepin strains of *C. militaris* were obtained. The genome and transcriptomic analyses revealed the following: (1) The CMS19 genome had a size of 30.96 Mb with 8 contigs and contained 32 detected secondary metabolite gene clusters, surpassing the published genomes of Cm01 and AMS333216, both predicting 28 clusters. This suggests that CMS19 can be used to produce diverse new natural products. (2) The phylogenetic and genetic differentiation analyses revealed a close relationship between *C. militaris* and *B. bassiana*. Genetic variation was observed among 64 strains from different geographical regions. Most cultivated and wild strains can be distinguished into their respective groups, respectively. Notably, the cultivated strains, CMCAD05, CMCAD27, and CMCLY01, exhibited close genetic proximity to the wild strains, suggesting recent artificial cultivation following domestication with a short domestication period. (3) We elucidated a hypothetical cordycepin metabolism pathway, initiating from the glycolysis and pentose phosphate and concluding at purine metabolism. In this metabolic pathway, we identified 12 out of 24 genes that had high upregulation levels, 9 genes that were significantly upregulated in high-cordycepin content stages. Meanwhile, we validated the expression pattern of key genes (Cns1–Cns4), which are consistent with previous research results [6]. This suggested that cordycepin synthesis was governed by a highly intricate metabolic network and not by one or a few genes. The genomic data and analytical results generated in this study would provide valuable genetic resources for *Cordyceps* evolution, genetic differentiation, and cordycepin synthesis. Furthermore, it can provide essential germplasm resources and theoretical support for the development of commercial varieties, genetic breeding research, and the extraction of functional products.

## Figures and Tables

**Table 1:** Summary of genome assembly and annotation of *C. militaris*.

Species Information	*C. militaris*
Total counts of scaffold sequences:	8
Total length of scaffold sequences:	30,963,950 bp
Scaffold N50	4,735,179 bp
Scaffold N90	1,818,051 bp
GC content (%)	52.69
N Length	0 bp
N content (%)	0
Gene Number	9781
Gene density	0.3
Gene average length	1527
Exon number per Gene	2.84
Exon average length	537
Exon GC percent (%)	58.92

**Table 2:** Secondary metabolic gene clusters in different *C. militaris* genomes.

Type	Genome Strain Name		
	CMS19	AMS333216	CM01
T1PKS	8	6	6
NRPS	7	7	7
NRPS-like	5	6	4
Terpene	4	3	4
T1PKS, NRPS	2	3	2
NRPS, T1PKS	4	2	4
indole	1	0	0
fungal-RiPP	1	0	0
NRPS, NRPS-like	0	1	1
Total	32	28	28

**Table 3:** qRT-PCR validation of differentially expressed genes.

Genes	Annotation	Log2FC	2^−△△ct^
*g5449*	adenylate kinase	0.78	0.63
*g3376*	adenosine kinase	1.77	1.22
*g4827*	oxidoreductase domain-containing protein	1.38	1.13
*g4826*	Phosphoribosyl aminoimidazole carboxylase	0.72	0.57
*g4825*	adenylate kinase	0.70	1.05
*g4824*	ABC multidrug transporter	−0.19	0.55
*g9123*	adenylate kinase	−1.50	0.16
*g6539*	adenylosuccinate lyase	0.74	0.71
*g1229*	adenylosuccinate synthetase	1.94	4.03
*g2310*	adenylosuccinate synthetase	−2.55	0.03
*g2322*	hypothetical protein	2.19	1.88
*g2847*	adenosine deaminase	1.46	1.34
*g3195*	adenosine deaminase	0.85	0.49

## Data Availability

We confirm that our experimental data are accurate, which supports the results and conclusions of this study. In addition to the data reflected in this article and its attachments, some of the data have been shared in the figshare database with the Doi link (https://www.figshare.com (accessed on 12 April 2024)) for reference.

## Supplementary Materials

The following supporting information can be downloaded at: https://www.mdpi.com/article/10.3390/genes15050626/s1: Figure S1. Repetitive sequence analysis of *C. militaris*; Table S1. Information description of *C. militaris*; Table S2. Genome annotation results of *C. militaris*; Table S3. Types of inferred secondary metabolite gene clusters and their distribution on chromosomes; Table S4. Prediction of gene clusters for secondary metabolite; Table S5. Resequencing data of 64 of *C. militaris* strains; Table S6. SNP detection and annotation of *C. militaris* strains; Table S7. Statistics on genes and differential expression of hypothetical Cordycepin metabolism.
