# Supplementary figures and images for: Genomic and Transcriptome Analysis Reveals the Biosynthesis Network of Cordycepin in Cordyceps militaris
Source: Genes (Basel). 2024 May 15;15(5):626. doi: 10.3390/genes15050626 (PMC11120935; doi:10.3390/genes15050626)

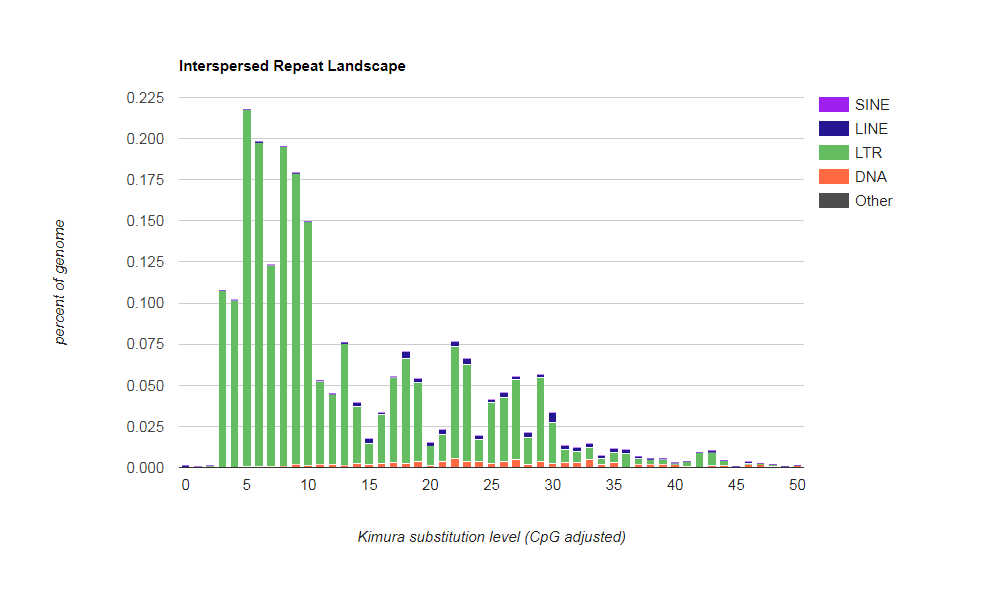

Supplement: Supplementary file 1 [file genes-15-00626-s001.zip › Figure S1. Repetitive sequence analysis of Cordyceps militaris.png]
